# Exploring the Psychological Effects of COVID-19 Home Confinement in China: A Psycho-Linguistic Analysis on Weibo Data Pool

**DOI:** 10.3389/fpsyg.2021.587308

**Published:** 2021-06-03

**Authors:** Peijing Wu, Nan Zhao, Sijia Li, Zeyu Liu, Yilin Wang, Tianli Liu, Xiaoqian Liu, Tingshao Zhu

**Affiliations:** ^1^CAS Key Laboratory of Behavioral Science, Institute of Psychology, Beijing, China; ^2^Department of Psychology, University of Chinese Academy of Sciences, Beijing, China; ^3^Institute of Population Research, Peking University, Beijing, China

**Keywords:** home confinement, mental health, COVID-19, psycho-linguistic analysis, LIWC, activity restriction

## Abstract

**Backgrounds:**

With the rapid spread of COVID-19, strict home confinement has been implemented in most parts of Chinese regions. Millions of people were not allowed to leave their homes except for special reasons. Home confinement plays an essential role in curbing pandemic and promoting preventive behaviors, but it may affect individuals’ mental health as well.

**Objects:**

The objective of this study was to explore the psychological impacts of home confinement.

**Materials and Methods:**

We collected more than 150,360 Weibo messages from 5,370 Chinese active users, and then extracted psycho-linguistic features from these messages. Psycho-linguistic analysis was carried out using the 2 (confinement vs. non-confinement) × 2 (before vs. after confinement) repeated measure analysis of variance (RM ANOVA).

**Results:**

The results showed that the frequency of positive emotion words was remarkably decreased during home confinement [*F*_(1,5368)_ = 7.926, *p* = 0.005, η^2^ = 0.001]. In high-endemic subgroup, home confinement also reduced the frequency of exclusion words [*F*_(1,3445)_ = 4.518, *p* = 0.034, η^2^ = 0.001] and inhibition words [*F*_(1,3445)_ = 10.154, *p* = 0.001, η^2^ = 0.003].

**Conclusion:**

Home confinement caused a decline in the use of positive emotion words. This indicates that home confinement can increase the frequency of negative emotions. The changes of exclusion words and inhibition words in high-endemic areas may be related to the high epidemic threat and the urgent need for social distancing in these areas.

## Introduction

At the end of 2019, an outbreak of novel coronavirus (COVID-19) has quickly spread in China (Huang C. et al., 2020). To control the epidemic, home confinement of varying strictness—from checkpoints at building entrances to hard limits on going outdoors—have been implemented for millions of people in China (NewYorkTimes, 2020). The details of such regulations might be slightly different from city to city, but they all limited daily activities of people.

Such quarantine tends to cause negative psychological effects (Brooks et al., 2020), especially in terms of emotion and cognition. The emotional symptoms include moody (Lee et al., 2005), fear (Desclaux et al., 2017; Caleo et al., 2018), anger (Cava et al., 2005; Caleo et al., 2018), anxiety (Bai et al., 2004), and depression (Hawryluck et al., 2004). The cognitive symptoms include confusion (Cava et al., 2005; Pan et al., 2005; Braunack-Mayer et al., 2013; Caleo et al., 2018), numbness (Pan et al., 2005), avoidance and high-risk judgment related to post-traumatic stress disorder (Bai et al., 2004; Reynolds et al., 2008; Wu et al., 2009). Previous studies of medical staff during SARS showed that being quarantined was a predictor of posttraumatic stress (Bai et al., 2004; Wu et al., 2009) and depression symptoms (Liu et al., 2012) in hospital employees. Another study investigated on the psychosocial responses of children and their parents during SARS and H1N1 found that quarantine and isolation could be offensive to a significant portion of children and their parents (Sprang and Silman, 2013).

Based on above evidences, we can infer that large-scale home confinement during COVID-19 is also likely to have a negative psychological impact on public. However, previous researches generally focus on special population at a small sample size, such as patients and medical staff. In addition, although there are precedents for similar activity restrictions, such as in areas of Singapore, Canada and China during severe acute respiratory syndrome (SARS) (Gupta et al., 2005; Hsieh et al., 2005; Ooi et al., 2005). But the difference is that restrictions in these precedents only isolate suspected cases and contacts within a limited range, while COVID-19 home confinement restricted daily activities of millions of normative population, regardless of whether they have been in contact with suspected patients. In order to reduce the risk of such undifferentiated isolation in terms of public mental health, and to provide references for future interventions, it is necessary to understand the potential psychological changes caused by COVID-19 home confinement.

To explore the psychological changes caused by public emergencies, the pretest-posttest research design is the most commonly practiced (Peterson and Seligman, 2003; Li et al., 2020; Su et al., 2020). Psychological states are usually measured by retrospective questionnaires, yet it may not be appropriate to measure the psychological effect of home confinement during COVID-19. Since home confinement was carried out in an emergency condition, it would have been impossible to predict the time of implementation. Hence, we could not possibly conduct any prior measurement. In addition, because home confinement might have been cancelled in cities where the epidemic was rapidly controlled in short time (e.g., only 1 week in Ningbo, Zhejiang), there would have been little time available to conduct any timely survey. For the above reasons, we considered other novel methods to explore the psychological impact of home confinement.

Due to the widespread use of the Internet and rapid growth of virtual environments, individuals spend increasingly more time online, especially on social media. Several studies have shown that public mental states can be effectively identified by analyzing the psycho-linguistic features obtained from Social Network Services (SNS), including Twitter, Weibo, etc. (Golder and Macy, 2011; Zhao et al., 2016; Alizadeh et al., 2019). For instance, Niu et al. (2014) found that psycho-linguistic features have obvious advantages in identifying emotions of Weibo users; Golder et al. (Golder and Macy, 2011) revealed the trend of positive and negative emotions of Twitter users by calculating word frequencies according to the Linguistic Inquiry and Word Count (LIWC); Alizadeh et al. (2019) analyzed the moral aspirations of political extremists on Twitter by word frequencies related to moral foundation. During the COVID-19 period, researchers also used psycho-linguistic features to explore the related psychological effects (Huang F. et al., 2020; Li et al., 2020; Su et al., 2020). Su et al. (2020) used LIWC to examine the impact of COVID-19 lockdown in Wuhan and Lombardy. Li et al. (2020) found some negative psychological effects caused by COVID-19 Epidemic Declaration by LIWC. Huang F. et al. (2020) revealed the roles of fear and collectivism in COVID-19 prevention based on the related psycho-linguistic features. Compared to conventional psychological surveys, psycho-linguistic features on social media have the advantages of traceability and real-time (Bollen et al., 2009; Golder and Macy, 2011), which can be matched with the time period of home confinement in various regions and effectively reflect the psychological changes of users (Tausczik and Pennebaker, 2010; Zhao et al., 2016). Therefore, we aimed to use the psycho-linguistic features on social media as a real-time measurement across large time span and diverse populations for determining the psychological impact of home confinement.

In this study, we aimed to explore the possible mental impacts of home confinement by analyzing the changes in psycho-linguistic features, extracted by the LIWC tool, among Chinese Weibo users. It is hypothetically assumed that home confinement may cause some negative psychological effects, which leads to a significant difference in relevant psycho-linguistic features before and after the confinement. Our findings can lay a foundation for further research on the psychological impacts of quarantine during COVID-19 pandemic, especially data supplements for large samples of a general population. Once the possible psychological effects were clarified, managers could formulate more effective and targeted public mental health policies. For example, if we found that home confinement had led to a decrease in the use of positive emotion words, managers could pay more attention on how to restore the public positive emotion, instead of focusing on public panic or anger. And researchers could further study what factors of home confinement reduced public positive emotion, so as to achieve more targeted intervention suggestions.

## Materials and Methods

### Data Collection

According to the National Coronary Pneumonia Epidemic Prevention and Control Headquarters’ announcements, 17 cities had implemented home confinement from January 23, 2020 to March 30, 2020. As shown in Table 1, we defined the above 17 cities as home confinement cities, while others as non-home confinement cities.

**TABLE 1:** Home confinement cities and their start and end time.

**Confinement cities**	**Start time**	**End time**
Wuhan, Hubei	February 11th	Unpublished
Huanggang, Hubei	February 1st	Unpublished
Ezhou, Hubei	February 4th	March 25th
Huangshi, Hubei	February 17th	March 23rd
Xiaogan, Hubei	February 14th	Unpublished
Wenzhou, Zhejiang	February 1st	February 16th
Taizhou, Zhejiang	February 2nd	February 13th
Yiwu, Zhejiang	February 4th	February 12th
Ningbo, Zhejiang	February 4th	February 10th
Bengbu, Anhui	February 3rd	March 23rd
Huaibei, Anhui	February 3rd	Unpublished
Fuyang, Anhui	February 7th	March 31st
Zhongshan, Guangdong	February 2nd	March 27th
Fangchenggang, Guangxi	February 3rd	Unpublished
Guigang, Guangxi	February 4th	Unpublished
Nanyang, Henan	February 4th	Unpublished
Songyuan, Jilin	February 3rd	March 20th

*“Confinement cities” are cities that issued a formal official notice to announce the implementation of home confinement between January 23, 2020, and March 30, 2020; The “Start time” and “End time” are the exact start and end time of home confinement, respectively.*

Next, we obtained samples from the public Weibo data pool composed of 1.16 million users (Li et al., 2014). This database regularly updates Weibo user profile information and the latest Weibo text content through crawler technology. First, we filtered the samples and extracted the valid user data set *S* according to the following criteria: (i) non-public, non-Chinese, non-commercial accounts; and (ii) published at least ten original Weibo posts from January 11 to February 21, 2020. Given that the cultural background and pandemic status of non-Chinese users may vary from those of Chinese users, we excluded them. Sociodemographic information of the selected participants are presented in Table 2. Privacy protection was strictly conducted, in compliance with the ethical principles listed previously (Kosinski et al., 2015). No participants consent was required during the study process. The research protocol was approved by the Ethics Committee of the Institute of Psychology, Chinese Academy of Sciences (approval number: H15009).

**TABLE 2:** Demographic characteristics of the selected participants.

	**All home confinement cities**	**High-endemic subgroup**	**Low-endemic subgroup**	**Non-confinement cites**
	**n (%)**	**n (%)**	**n (%)**	**n (%)**
**Sex**				
Male	480 (26.82)	320 (27.85)	160 (24.96)	960 (26.82)
Female	1,310 (73.18)	829 (72.15)	481 (75.04)	2,620 (73.18)
**Age**				
18–30	255 (14.25)	154 (13.40)	101 (15.76)	464 (12.96)
31–40	128 (7.15)	78 (6.79)	50 (7.80)	249 (6.96)
≥ 41	18 (1.00)	11 (0.96)	7 (1.09)	46 (1.28)
Missing data	1,389 (77.60)	906 (78.85)	483 (75.35)	2,821 (78.80)
**Regions of China**				
Eastern China	469 (26.20)	0 (0)	469 (73.17)	2,836 (79.22)
Central China	1,271 (71.01)	1,149 (100)	122 (19.03)	2,40 (6.70)
Western China	50 (2.79)	0 (0)	50 (7.80)	504 (14.08)
Total	1,790 (100)	1,149 (100)	641 (100)	3,580 (100)

After that, we identified the confinement user set *S*_lock_ in which the location was one of the 17 confinement cities and the non-confinement user set *S*_non−lock_ = *S*−*S*_lock_. For each confinement city *C*_*i*_(*i* = 1.17), we set the 2 weeks before and after home confinement as *T*_before,*i*_ and *T*_after,*i*_, and screened out the participants of confinement group *E*_*i*_ and non-confinement group *F*_*i*_ according to the following procedures:

i) For each Weibo user in *S*_lock_, if its location was *C*_*i*_ and at least one Weibo was posted every day in average between *T*_before,*i*_ and *T*_after,*i*_, the user was included in the confinement group *E*_*i*_.
ii) The numbers of male users *E*_M,*i*_ and female users *E*_F,*i*_ in the confinement group *E*_*i*_ were counted.
iii) The *E*_M,i_*2 male users and *E*_F,i_*2 female users in *S*_non–lock_ who posted at least one Weibo every day in average between *T*_before,*i*_ and *T*_after,*i*_ were randomly selected and included in the non-confinement group *F*_*i*_.

Through the above process, the ratio of participant numbers between non-confinement and confinement groups reached 2:1. Such setting could increase the statistical power since the number in confinement group was much lesser than another group (Lydersen, 2018). Considering that the durations of home confinement in Taizhou, Ningbo and Yiwu were too short (<2 weeks), the users in these 3 cities were excluded from subsequent analysis. All the above processes were carried out based on the users’ profile information available on Weibo. It shall be emphasized that the users’ location information was also obtained from their registration profile. This might not be the exact geographical location, but it was related to the user. Lastly, for all selected users, we collected their Weibo posts within 2 weeks before and after the start time of home confinement in their own city. A total of 150,360 Weibo posts were collected.

### Data Analysis

After eliminating the forwarding Weibo posts, TextMind system 4.0^1^ (a Chinese segmentation and word frequency statistical tool) was used to extract the psycholinguistic features (Gao et al., 2013). This system analyzed the input text in Chinese, and then employed Simplified Chinese LIWC (SCLIWC) dictionary (Zhao et al., 2016) for word frequency statistics.

The SCLIWC dictionary has been proven to detect individuals’ attentional focus, emotionality and thinking styles effectively (Tausczik and Pennebaker, 2010; Zhao et al., 2016). The words have previously been categorized into over 80 linguistic dimensions, including psychological processes (e.g., positive and negative emotion categories, cognitive processes such as the use of causation words), relativity-related words (e.g., time, verb tense, motion, space), and traditional content dimensions (e.g., sex, death, home, occupation) (Pennebaker and Lay, 2002). A recent study has shown that similar quarantine tends to cause negative emotions and cognition effects (Brooks et al., 2020). Hence, the affective processes words and cognitive words (13 categories in total) were selected as the affective and cognitive linguistics features of Weibo users, respectively. Examples on each word category are summarized in Table 3.

**TABLE 3:** Examples on affective processes words and cognitive words in SCLIWC.

**Category**	**Example**
**Affective process words**	
Positive emotion	Love, nice, sweet
Negative emotion	pain, harmful, worst
Anxiety	Stress, concern, frightening
Anger	Accuse, fury, curse
Sad	Lose, cry, heart-broken
**Cognitive process words**	
Insight	Understand, essence, discuss
Cause	Why, because, conclusion
Discrepancy	More, less, can’t
Tentative	Hesitation, options, doubt
Certain	Guarantee, obvious, doomed
Inhibition	Suppression, confinement, manage
Inclusive	Lock, constrain, stop
Exclusive	But, without, exclude

Given that this study was in the background of pandemic and the severity of prior epidemic also greatly influenced people’s mental states (Murray and Schaller, 2012), we divided all the home confinement cities into two subgroups: high-endemic subgroup and low-endemic subgroup. Since Hubei had more than 83% of confirmed cases in China (as of March 1, 2020), all cities in this province were defined as high-endemic areas, while the remaining were low-endemic areas. For all home confinement cities group, high-endemic subgroup and low-endemic subgroup, the analysis was conducted separately. The entire sample filtering and grouping process is shown in Supplementary Figure 1, and the demographic profiles of the selected participants are listed in Table 2.

RM ANOVA was performed on the frequencies of each word using SPSS 24.0 (Statistical Product and Service Solutions), with home confinement status (confinement vs. non-confinement) as the between-subject variable and time (before vs. after confinement) as the within-subject variable. To exclude possible differences in pandemic severity between confinement and non-confinement groups, we only focused on the variables with significant interactions. The significant interaction meant that the difference between groups was inconsistent before and after home confinement, and this difference was mainly caused by home confinement.

## Results

Several significant interactions of group (confinement vs. non-confinement) ^∗^ time (before vs. after confinement) were found through RM ANOVA. As these interactions reflected the impacts of home confinement on psycho-linguistic features, Simple Effect Analysis was carried out subsequently.

### All Home Confinement Cities

**FIGURE 1:**
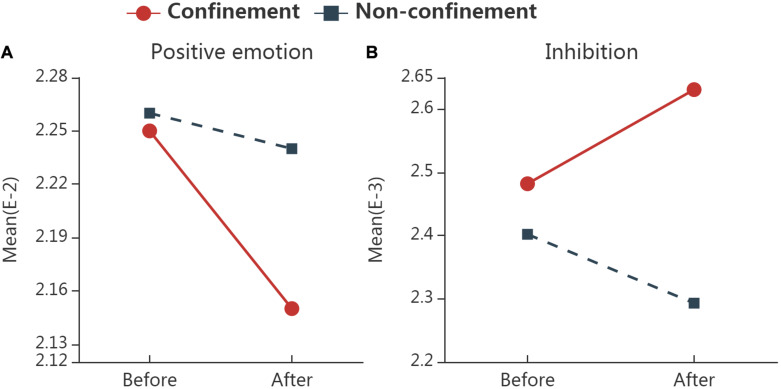
The interaction plots for words with significant interactions in the group of all home confinement cities. Significant group (confinement vs. non-confinement) * time (before vs. after confinement) interaction was found in the frequency of positive emotion words **(A)** and Inhibition words **(B)** by RM ANOVA. “Confinement” represents samples in all 14 cities where home confinement have implemented; “Non-confinement” represents samples that have not experienced home confinement; “Before” represents the data collected from 2 weeks before home confinement, and “After” represents that from 2 weeks after.

The results of RM ANOVA in the group of all home confinement cities are summarized in Table 4. The group × time interaction was significant for the frequency of positive emotion words (e.g., love, nice, sweet) [*F*_(1,5368)_ = 7.926, *p* = 0.005, η^2^ = 0.001] and inhibition words (e.g., lock, constrain, stop) [*F*_(1,5368)_ = 10.166, *p* = 0.001, η^2^ = 0.002]. Figure 1 reveals the interaction plots for all home confinement cities groups. Further Simple Effect Analysis showed that the frequency of positive emotion words was decreased in the confinement group [*F*_(1)_ = 16.173, *p* < 0.001, η^2^ = 0.003], and was remarkably lower than that in the non-confinement group after home confinement [*F*_(1)_ = 7.569, *p* = 0.006, η^2^ = 0.001]. The frequency of inhibition words was increased in the confinement group [*F*_(1)_ = 5.066, *p* = 0.024, η^2^ = 0.001], and was markedly higher than that in the non-confinement group after home confinement [*F*_(1)_ = 26.522, *p* < 0.001, η^2^ = 0.005].

**TABLE 4:** The results of RM ANOVA in all home confinement cities.

	**Confinement (*n* = 1,790)**				**Non-confinement (*n* = 3,580)**				
	**Before**		**After**		**Before**		**After**		*F*_group__*__time_
	**M (E-3)**	**SD (E-3)**	**M (E-3)**	**SD (E-3)**	**M (E-3)**	**SD (E-3)**	**M (E-3)**	**SD (E-3)**	
**Affective process words**									
Positive emotion	22.53	11.71	21.52	10.53	22.6	12.71	22.53	12.72	7.926**
Negative emotion	9.58	5.44	9.72	5.65	9.09	5.46	9.15	5.85	0.149
Anxiety	1.27	1.56	1.26	1.59	1.18	1.60	1.11	1.45	1.136
Anger	2.39	2.22	2.27	2.14	2.32	2.15	2.25	2.19	0.315
Sad	1.64	1.75	1.58	1.74	1.53	1.76	1.48	1.75	0.089
**Cognitive process words**									
Insight	7.73	4.45	8.02	4.49	7.51	4.71	7.88	4.85	0.285
Cause	4.47	3.10	4.72	3.27	4.43	3.28	4.68	3.52	0.001
Discrepancy	12.4	6.27	12.1	6.54	11.9	6.28	11.4	6.66	0.605
Tentative	9.16	5.30	9.35	5.63	8.69	5.41	8.87	5.66	0.002
Certain	8.02	4.05	7.90	4.33	7.63	4.23	7.37	4.19	0.945
Inhibition	2.48	2.08	2.63	2.47	2.40	2.29	2.29	2.16	10.166***
Inclusive	19.3	8.24	18.7	8.57	18.7	8.81	18.2	8.54	0.055
Exclusive	9.28	5.24	9.64	5.54	8.94	5.46	9.04	5.50	2.657

“Confinement” represents the samples in all 14 cities where home confinement has implemented; “Non-confinement” represents the samples that have not experienced home confinement; “Before” represents the data collected at 2 weeks before home confinement, and “After” represents the data collected at 2 weeks after home confinement. M, mean; SD, standard deviation; F_group*time_ represents the F-value of RM ANOVA. *p < 0.05, **p < 0.01, and ***p < 0.001.

### High-Endemic Subgroup

**FIGURE 2:**
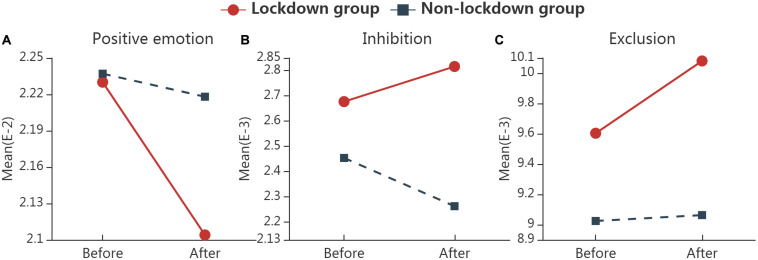
The interaction plots for words with significant interactions in high-endemic subgroup. Significant group (confinement vs. non-confinement) * time (before vs. after confinement) interaction was found in the frequency of positive emotion words **(A)**, Inhibition words **(B)**, and Exclusion words **(C)** by RM ANOVA. “Confinement” represents samples in all 14 cities where home confinement have implemented; “Non-confinement” represents samples that have not experienced home confinement; “Before” represents the data collected from 2 weeks before home confinement, and “After” represents that from 2 weeks after.

The results of RM ANOVA in the subgroup of high-endemic cities are presented in Table 5. The group × time interaction was significant for the frequency of positive emotion words [*F*_(1,3445)_ = 6.903, *p* = 0.009, η^2^ = 0.002], inhibition words [*F*_(1,3445)_ = 10.154, *p* = 0.001, η^2^ = 0.003] and exclusive words (e.g., but, without, exclude) [*F*_(1,3445)_ = 4.518, *p* = 0.034, η^2^ = 0.001]. Figure 2 shows the interaction plots for high-endemic subgroups. Further simple effect analysis revealed that the frequency of positive emotion words was decreased in the confinement group [*F*_(1)_ = 15.060, *p* < 0.001, η^2^ = 0.004], and was remarkably lower than that in the non-confinement group after home confinement [*F*_(1)_ = 10.337, *p* = 0.001, η^2^ = 0.003]. The frequency of inhibition words was increased in the confinement group [*F*_(1)_ = 2.703, *p* = 0.100, η^2^ = 0.002], and was markedly higher than that in the non-confinement group before [*F*_(1)_ = 7.367, *p* = 0.007, η^2^ = 0.013] and after home confinement [*F*_(1)_ = 44.703, *p* < 0.001, η^2^ = 0.013]. The frequency of exclusion words was increased in the confinement group [*F*_(1)_ = 8.029, *p* = 0.005, η^2^ = 0.002], and was apparently higher than that in the non-confinement group before [*F*_(1)_ = 8.776, *p* = 0.003, η^2^ = 0.003] and after home confinement [*F*_(1)_ = 26.311, *p* < 0.001, η^2^ = 0.008].

**TABLE 5:** The results of RM ANOVA in high-endemic subgroup.

	**Confinement (*n* = 1149)**				**Non-confinement (*n* = 2298)**				
	**Before**		**After**		**Before**		**After**		*F*_group__*__time_
	**M (E-3)**	**SD (E-3)**	**M (E-3)**	**SD (E-3)**	**M (E-3)**	**SD (E-3)**	**M (E-3)**	**SD (E-3)**	
**Affective process words**									
Positive emotion	22.05	11.86	20.85	9.77	22.37	12.70	22.17	12.10	6.903**
Negative emotion	10.1	5.06	9.98	5.55	9.27	5.41	9.11	5.93	0.071
Anxiety	1.29	1.50	1.32	1.62	1.17	1.63	1.10	1.50	1.651
Anger	2.57	2.26	2.31	2.13	2.34	2.13	2.24	2.18	2.49
Sad	1.80	1.76	1.60	1.60	1.59	1.82	1.48	1.82	1.306
**Cognitive process words**									
Insight	7.96	4.30	8.21	4.24	7.49	4.79	8.05	5.06	2.515
Cause	4.52	3.02	4.73	3.00	4.34	3.32	4.74	3.57	1.895
Discrepancy	12.7	5.92	12.13	5.91	11.81	6.39	11.31	6.49	0.233
Tentative	9.41	4.95	9.50	5.35	8.78	5.56	8.98	5.86	0.242
Certain	8.16	3.77	7.99	4.01	7.64	4.29	7.37	4.21	0.392
Inhibition	2.68	2.13	2.82	2.53	2.45	2.35	2.26	2.16	10.154***
Inclusive	18.9	7.36	19.02	8.22	17.92	8.22	18.21	8.66	0.417
Exclusive	9.60	5.10	10.13	5.44	9.02	5.56	9.06	5.54	4.518*

“Confinement” represents the samples in all 14 cities where home confinement has implemented; “Non-confinement” represents the samples that have not experienced home confinement; “Before” represents the data collected at 2 weeks before home confinement, and “After” represents the data collected at 2 weeks after home confinement. M, mean; SD, standard deviation; F_group*time_ represents the F-value of RM ANOVA. *p < 0.05, **p < 0.01, and ***p < 0.001.

### Low-Endemic Subgroup

**FIGURE 3:**
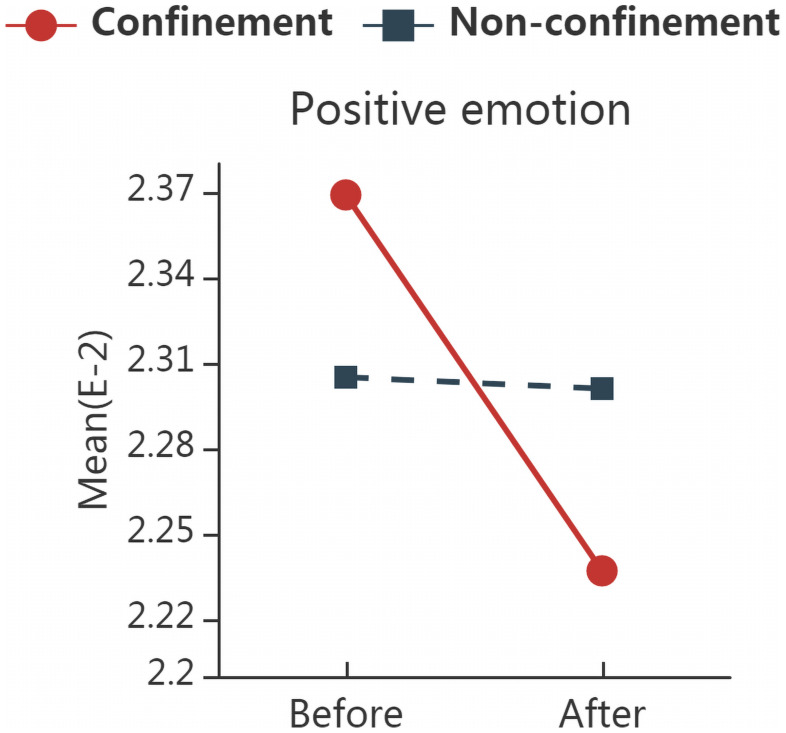
The interaction plots for words with significant interactions in low-endemic subgroup. Significant group (confinement vs. non-confinement) * time (before vs. after confinement) interaction was found in the frequency of positive emotion words by RM ANOVA. “Confinement” represents samples in all 14 cities where home confinement have implemented; “Non-confinement” represents samples that have not experienced home confinement; “Before” represents the data collected from 2 weeks before home confinement, and “After” represents that from 2 weeks after.

Based on the analysis of the low-endemic areas, only positive emotion words had a significant group × time interaction [*F*_(1,1921)_ = 5.796, *p* = 0.016, η^2^ = 0.003]. The results are demonstrated in Table 6. Figure 3 shows the interaction plots for the low endemic subgroup. Further simple effect analysis indicated that the frequency of positive emotion words was decreased in the confinement group [*F*_(1)_ = 9.374, *p* = 0.002, η^2^ = 0.005], and no significant difference was observed between confinement and non-confinement groups before and after home confinement.

**TABLE 6:** The results of RM ANOVA in low-endemic subgroup.

	**Confinement (*n* = 641)**				**Non-confinement (*n* = 1282)**				
	**Before**		**After**		**Before**		**After**		*F*_group__*__time_
	**M (E-3)**	**SD (E-3)**	**M (E-3)**	**SD (E-3)**	**M (E-3)**	**SD (E-3)**	**M (E-3)**	**SD (E-3)**	
**Affective process words**									
Positive emotion	23.73	12.41	22.41	11.72	23.11	12.71	23.01	13.72	5.796*
Negative emotion	8.70	5.96	9.25	5.79	8.76	5.52	9.22	5.69	0.083
Anxiety	1.23	1.67	1.17	1.54	1.20	1.55	1.14	1.34	0.001
Anger	2.07	2.11	2.19	2.16	2.30	2.19	2.26	2.21	1.598
Sad	1.36	1.68	1.54	1.97	1.42	1.66	1.50	1.64	1.272
**Cognitive process words**									
Insight	7.33	4.68	7.67	4.90	7.55	4.58	7.57	4.42	1.619
Cause	4.38	3.26	4.72	3.71	4.59	3.22	4.56	3.43	3.452
Discrepancy	11.84	6.82	12.01	7.53	12.12	6.07	11.62	6.95	3.488
Tentative	8.73	5.85	9.09	6.11	8.52	5.12	8.67	5.29	0.606
Certain	7.78	4.52	7.73	4.86	7.60	4.10	7.36	4.15	0.603
Inhibition	2.13	1.94	2.30	2.32	2.31	2.18	2.35	2.17	1.019
Inclusive	20.02	9.59	18.22	9.15	20.12	9.63	18.13	8.30	0.13
Exclusive	8.69	5.45	8.85	5.63	8.78	5.27	8.99	5.42	0.03

“Confinement” represents the samples in all 14 cities where home confinement has implemented; “Non-confinement” represents the samples that have not experienced home confinement; “Before” represents the data collected at 2 weeks before home confinement, and “After” represents the data collected at 2 weeks after home confinement. M, mean; SD, standard deviation; F_group*time_ represents the F-value of RM ANOVA. *p < 0.05, **p < 0.01, and ***p < 0.001.

## Discussion

This study used Weibo data and psycho-linguistic analysis to explore the psychological impacts of home confinement. Our results found a decline in the frequency of positive emotion words (e.g., love, nice, sweet) after home confinement in all home confinement cities group, high-endemic subgroup and low-endemic subgroup. This indicates that home confinement is associated with an increase in the frequency of negative emotions. Compared with the low-endemic subgroup, the high-endemic subgroup reported more diverse changes such as the increased use of exclusion (e.g., but, without, exclude) and inhibition (e.g., lock, constrain, stop) words.

### The Association Between Home Confinement and Decreased Positive Emotions

The frequency of positive emotion words (e.g., love, nice, sweet) decreased after home confinement in all three groups, indicating that home confinement is associated with a decline in people’s positive emotions (Kahn et al., 2007; Golder and Macy, 2011) regardless of high-endemic or low-endemic status. There could be many reasons for the decline in positive emotions. On the one hand, home confinement limited the opportunities to experience various sources of positive emotion, including the access to nature (Johnsen, 2014), social gatherings (House et al., 1988) and outdoor sports (Coon et al., 2011). On the other hand, growing evidence has suggested that people who stay at home for a long time may have a low level of self-satisfaction (Wu et al., 2019), which in turn weakens their happiness and self-efficacy, and ultimately impairs their ability to perceive positive emotions (Bandura, 1977).

It is also worth noting that positive and negative emotions are not entirely negatively correlated (Clark et al., 1989). We found that home confinement was not significantly associated with increased frequencies of fear, anger or any other negative emotions. Moreover, our findings implied that home confinement mainly suppressed the positive emotions of the residents, but did not explicitly induce a specific negative emotion. It seems to be different from previous reports. Considering that previous researches mainly focused on specific sample groups such as patients and medical staff, they might have already borne more pressure from pandemic (Bai et al., 2004; Wu et al., 2009; Brooks et al., 2020). However, our research was primarily targeted at the general populations, especially individuals who had no close contact with COVID-19. Therefore, the negative psychological impact of home confinement might not be so significant. Besides, in the early stages of data collection (after January 23, 2020), public negative emotions, such as anxiety, fear and angry, had occurred at a very high level (Li et al., 2020). Thus, home confinement might not able to further increase the frequency of negative emotions.

### The Influence of Home Confinement on Cognition Processing

Inhibition words (e.g., lock, constrain, stop) and exclusion words (e.g., but, without, exclude) both reflect the cognitive processing characteristics of individuals (Zhao et al., 2016). Inhibition words represent the sense of being restricted and constrained (Pennebaker and Lay, 2002). There was no doubt that home confinement would have resulted in restrictions on daily activities such as shopping and outdoor sports. From the perspective of leisure constraints models (Crawford et al., 1991), these restrictions can be regarded as structural barriers. Our results indicated that home confinement also increased individuals’ sense of being constrained. As such, this could be considered as intrapersonal barriers and might further reduce the level of motivation and participation in leisure, thereby affecting their stress management abilities on both physical and psychological illness (Louise, 1995).

Exclusion words represent a measure of cognitive complexity, and the rising of it indicates the increase of cognitive complexity (Tausczik and Pennebaker, 2010). It is obvious that home confinement has introduced great changes in people’s lifestyles, and in this scenario, people need to consume more cognitive resources to deal with various restrictions, thus leading to the increase of cognitive complexity (Franconeri et al., 2013). Prior research has suggested that exclusion words are richer in genuine information than deceptive content such as rumors (Zhou et al., 2004; Chua and Banerjee, 2016). In this study, home confinement led to the increased use of exclusion words, indicating that people in this state are more seeking for genuine information.

Although the extents and type of activity restrictions between high-endemic and low-endemic areas are similar, we did not find a significant interaction for the use of inhibition words and exclusion words in low-endemic areas. This might be attributed to higher epidemic threats and stronger government publicity in high-endemic areas. To control the severe epidemic situation, local governments in high-endemic areas had made more considerable efforts to implement strict isolation procedures, which might amplify people’s sense of restrictions (Kasperson et al., 1988). What’ more, since the residents in high-endemic areas are facing serious disease threats, they are more likely to share some self-protection information (Pyzczynski et al., 2004). Abundant information and complex contents could increase cognitive complexity as well (Van Knippenberg et al., 2015). As a comparison, the frequency of exclusion words in low-endemic subgroup also showed an upward trend, but was not obvious compared to high-endemic subgroup.

## General Discussion

In summary, this study examined the psychological impacts of an extreme measure—home confinement in the pandemic outbreak situation. Our results showed that home confinement generally caused a decline in the use of positive emotion words, indicating that home confinement generally can increase the frequency of negative emotions.

A decline in positive emotion could bring some negative effects such as worsening the crisis stress response during the pandemic (Fredrickson et al., 2003) and impairing the performance at the workplace in the short time (Staw et al., 1994). Less long-term positive emotions might result in poorer physical and mental health (Richman et al., 2005; Cohen and Pressman, 2006). Recent findings (Brooks et al., 2020) demonstrated that quarantine could increase a wide range of negative psychological effects. A nationwide survey during COVID-19 highlight the high risk of psychological symptoms related to quarantine including anxiety, depression, insomnia, and acute stress (Wang et al., 2021). Our results complement those studies showing that an internal reason for these negative effects might be a decline in positive emotion.

For the decline of positive emotion, we could advocate corresponding measures to restore and increase the experience of positive emotions, such as natural contacts (Johnsen, 2014), meditation (Fredrickson et al., 2017), music (Croom, 2015), prosocial behavior (Varma et al., 2020), sports (Maher et al., 2021), in-person social interactions with friends (Lades et al., 2020), expressing gratitude and visualizing best possible selves (Sheldon and Lyubomirsky, 2006). Moreover, we could shorten the home confinement duration and encourage suitable outdoor activities. These activities might help to reduce the possibility of negative psychological effects such as depression and anxiety during COVID-19 (Lades et al., 2020).

Besides, our results also showed that home confinement led to the increased use of inhibition and exclusion words. This suggests that home confinement can promote the people’s sense of being constrained and cognitive complexity. People under confinement may feel confused since isolation and inadequate guidelines (Cava et al., 2005; Pan et al., 2005; Braunack-Mayer et al., 2013; Caleo et al., 2018). In response, public health authorities could provide sufficient and clear information as much as possible. Thus, ensuring the public have a good understanding of the disease in question and the reasons for home confinement should be a priority (Brooks et al., 2020).

In addition, our results revealed that the public’s response to home confinement was different in terms of cognition in high-endemic vs. low-endemic areas. These differences should be considered accordingly when developing anti-epidemic polices. To receive the best confinement effect and minimize the cost of confinement, the local authorities should pay more attention to the restrictions in different regions.

In conclusion, this study found that home confinement led to a decline in the use of positive emotion words, and led to an increase of the use of inhibition and exclusion words in high-endemic areas. At the theoretical level, we adopted a longitudinal study to compare the psychological characteristics of a large sample before and after confinement. While other studies only obtained self-reports of participants after isolation. Longitudinal design could help eliminate irrelevant interference factors such as recall errors. At the practical level, other studies often emphasize the importance of public mental health services. Our research put more emphasis on using the public’s own positive emotions to combat potential psychological risks. Such measures could give full play to the subjective initiative of the public and the intervention cost is relatively lower.

## Limitations and Future Work

There are some unavoidable limitations in our study. Firstly, there were few home confinements implemented at smaller scales, such as in a single community or even in one building. As the affected population was relatively small, the samples were excluded. Secondly, we could not obtain the health status of all subjects from the network data. However, in general, most of the active Weibo users were healthy individuals. Therefore, our conclusion was applicable to the public. Thirdly, our samples only included the active users of Weibo, and did not cover people who publish less than one post per day on average. Fourthly, the user’s registration might deviate from the user’s actual regional information, which may lead to slightly inaccurate results. Lastly, the emotions mentioned in this study were all explicit emotions. Implicit emotions were difficult to be measured directly from social media texts. Thus, more researches should be carried out in the future.

During the pandemic outbreaks, the public psychological state is an essential factor to consider, and it is important to understand the potential impact of home confinement on psychological state. This would be a starting point in exploring more profound effects, more relevant factors, such as other implicit emotions, and more precise methods that can be applied in future research. For example, we can use natural language processing (NLP) technology to analyze the implicit emotions in web texts. Most advanced methods can obtain 70∼87% accuracy (Alswaidan and Menai, 2020).

## Data Availability Statement

The raw data supporting the conclusions of this article will be made available by the authors, without undue reservation.

## Ethics Statement

This research project was approved by the Institute of Psychology of the Chinese Academy of Sciences’ Ethical Committee (project number: H15009). Written informed consent to participate in this study was provided by the participants’ legal guardian/next of kin.

## Author Contributions

NZ and TZ conceived and planned this article. PW and YW carried out the search. TZ collected and provided the data. PW analyzed the data and drafted the manuscript. YW collected some information. ZL and SL drafted the discussion part of the manuscript. NZ, TZ, TL, XL, and PW reviewed and edited the writing. All authors contributed to the article and approved the submitted version.

## Conflict of Interest

The authors declare that the research was conducted in the absence of any commercial or financial relationships that could be construed as a potential conflict of interest.

## Footnotes

1 http://ccpl.psych.ac.cn/textmind/

## Supplementary Material

Supplementary Figure 1 The flow chart of sample filtering and grouping process.
