# Supplementary figures and images for: Exploring the Psychological Effects of COVID-19 Home Confinement in China: A Psycho-Linguistic Analysis on Weibo Data Pool
Source: Front Psychol. 2021 Jun 3;12:587308. doi: 10.3389/fpsyg.2021.587308 (PMC8209261; doi:10.3389/fpsyg.2021.587308)

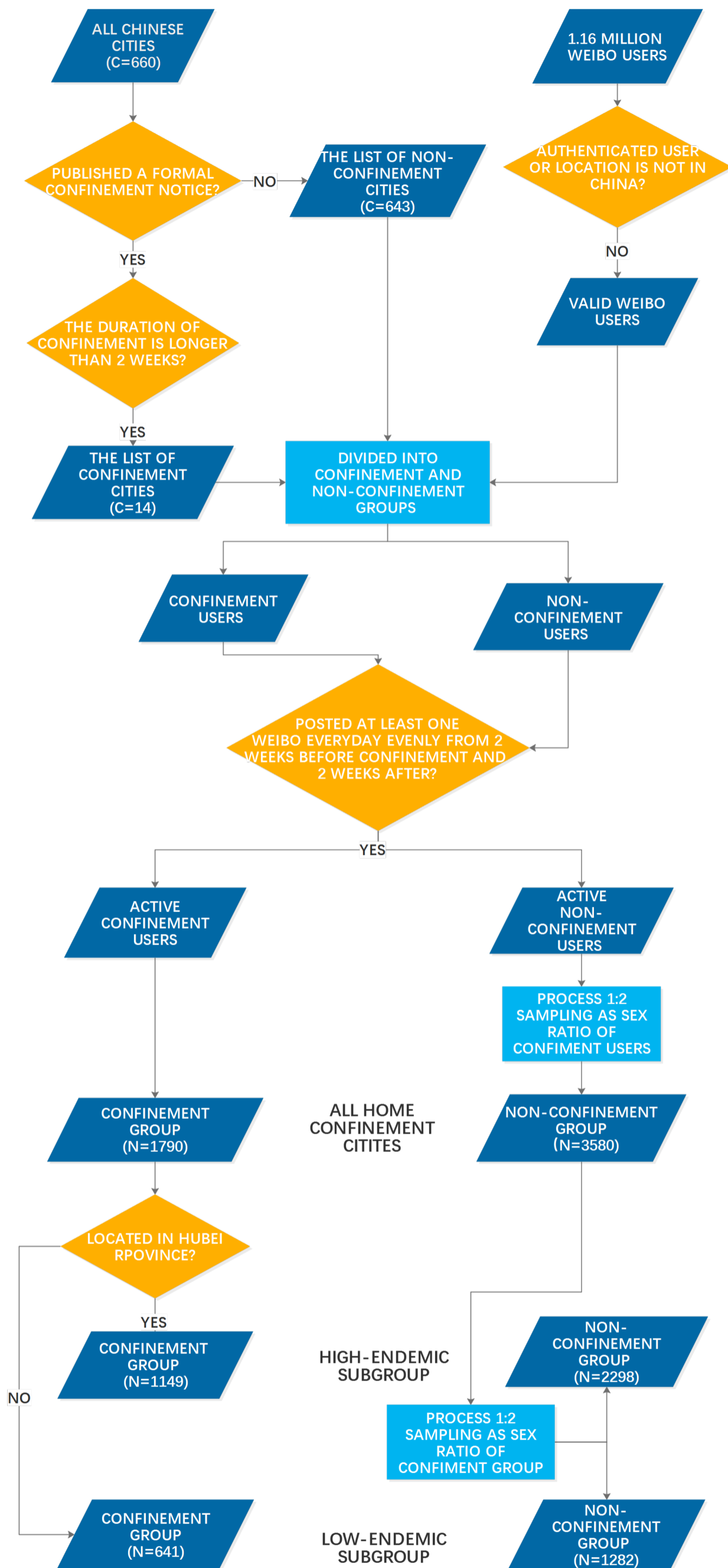

Supplement: Supplementary Figure 1 — The flow chart of sample filtering and grouping process. [file Data_Sheet_1.PDF]
